# RSM Optimization for the Recovery of Technofunctional Protein Extracts from Porcine Hearts

**DOI:** 10.3390/foods9121733

**Published:** 2020-11-25

**Authors:** Dolors Parés, Mònica Toldrà, Estel Camps, Juan Geli, Elena Saguer, Carmen Carretero

**Affiliations:** Institute for Food and Agricultural Technology (INTEA), University of Girona, Escola Politècnica Superior (EPS-1), C/Maria Aurèlia Capmany 61, 17003 Girona, Spain; monica.toldra@udg.edu (M.T.); estelcamps2@gmail.com (E.C.); juangeli97@gmail.com (J.G.); elena.saguer@udg.edu (E.S.); carmen.carretero@udg.edu (C.C.)

**Keywords:** meat byproducts, porcine heart, protein extraction, response surface methodology, technofunctional properties

## Abstract

Meat byproducts, such as the internal organs from slaughtered animals, are usually underutilized materials with low commercial value. The functional (emulsifying, gelling, and foaming) properties of soluble protein extracts derived from pork hearts were investigated, as well as their molecular weight distribution. A central composite design (CCD) for two process variables (pH and ionic strength of the extraction buffer) was used to foreknow the effects of the process conditions on the physicochemical characteristics and technofunctionality of the protein extracts by means of the response surface methodology (RSM). SDS-PAGE patterns of the heart protein solutions revealed multiple bands with molecular weights ranging from 15 to 220 kDa, mainly corresponding to sarcoplasmic, myofibrillar, as well as blood proteins. The best extraction conditions to obtain protein fractions with good foaming properties would correspond to acid pH (pH ≤ 5) and high salt content (2–4%). On the contrary, solutions recovered at pH > 5 with low NaCl contents were the ones showing better emulsifying properties. Regarding gelation ability, heat-induced gels were obtained from extracts at pH 6.5–8, which showed improved firmness with increasing NaCl content (2–4%). Satisfactory second-order polynomial models were obtained for all the studied response variables, which can be useful in guiding the development of functional ingredients tailored for specific uses to maximize applications.

## 1. Introduction

Efficient utilization of meat byproducts such as blood and offal is important for the profitability of the meat industry. It has been estimated that significant percentages of the gross income from food-producing animals come from these byproducts, about 11.4% and 7.5% from beef and pork, respectively [1]. Most byproducts have good nutritive value and can be utilized for food, to a greater or lesser degree depending on traditions, culture, and religion, as well as regulatory requirements. Traditional markets for edible meat byproducts have gradually decreased because of quality and health preoccupations; which have led to an increased focus on nonfood uses, such as pet foods, animal feed, pharmaceuticals and cosmetics [1,2], fertilizers, and biodiesel generation sources [3].

Currently, maximizing the use of animal proteins in the food industry is a crucial challenge due to the exponential increase in the meat protein demand and because of environmental concerns related to the meat chain. Thus, it is highly advisable to consider all byproducts as raw materials that can be changed into valuable food products or ingredients [4,5,6]. Such added value can be obtained in terms of shelf stability, more convenience, better sensory quality, and also improved technological functions, which can lead to the development of flavoring ingredients, water bonding agents, and stabilizers or emulsifiers [3].

Various categories of nonmeat technofunctional ingredients as such are chemicals, like sodium chloride, phosphates, carbonates and citrates; hydrocolloids, starches, flours, and vegetable fibers; or proteins like vegetable, dairy, and egg proteins; are used by meat processors to achieve different technological requirements and to meet consumer expectations. Some of these ingredients can be considered problematic to reach clean-label purposes or even may pose health concerns because they are catalogued as allergens. Thus, the development of alternative functional ingredients of meat origin can be a smart way to solve both aspects.

Meat byproducts can be a valuable source of technofunctional ingredients [2,6,7,8,9,10,11,12]. Among them, internal animal organs such are liver, lungs, brain, spleen, or heart, usually are low commercial valued and underutilized materials. Since they contain varying amounts of myofibrillar, sarcoplasmic, and stromal proteins when compared with those of lean meat [2,13,14], differences in the behavior of protein fractions from these byproducts when heated, as well as in the role of salts and pH on water holding capacity and texture, are conceivable. In this context, more information on the functional properties of protein fractions of meat byproducts can be useful for food processors.

The present work focuses on obtaining protein extracts from porcine hearts. Some studies on this topic can be found in the recent literature. Kim et al. (2017) tested the use of ultrasound-assisted extraction methods to extract proteins from myofibrils of porcine myocardium. They reported that sonicated samples produced higher protein extraction rates without the need for high salt concentrations [15]. Tsermoula et al. (2018) compared alkali (pH 11) and acid (pH 2) solubilization followed by isoelectric precipitation to prepare protein-rich extracts from bovine and porcine hearts. Both myofibril extracts showed good heat-induced gelling properties, which allowed for inferring its potential application as functional ingredients for processed meat products [16].

The objective of the present study was gain insight into the knowledge of technofunctional properties of porcine heart soluble proteins, aiming at maximizing their applications in the most efficient way. Both the raw material and the extraction conditions determine the kind and the amount of proteins that can be solubilized, as well as their structure or unfolding degree. All these factors may have a great influence on the functionality of the protein extracts obtained. Thus, we followed the same approach previously used in the obtaining of protein concentrates from porcine spleens [11]. Response surface methodology (RSM) was used to foreknow the effects of the extraction conditions (at different pH and salt concentration of the buffer solution) on the physicochemical characteristics and technofunctional properties of the soluble protein extracts derived from pork heart. The functional properties, such as solubility, gelling, emulsifying, and foaming properties, were determined.

## 2. Materials and Methods

### 2.1. Sample Collection and Preparation

Fresh hearts from recently slaughtered white pigs (LargeWhite × Landrace × Pietrain × Duroc commercial crossbred; 6 months old and 100 kg weight) were supplied by a local industrial slaughterhouse (Norfrisa S.A., Riudellots de la Selva, Girona, Spain) and were transported at 5 ± 1 °C to the laboratory. Firstly, the hearts were weighed and their pHs were measured in triplicate using a pH meter with a solid probe (LPG Crison 22, Barcelona, Spain). After being polished through removing fat and valves and blood vessels, hearts were divided into small pieces and kept refrigerated until they were analyzed or processed.

### 2.2. Experimental Design

The microbiological quality and physicochemical characterization of raw material were carried out on hearts collected in six different days but under the same conditions. Sample units for compositional analysis consisted of a mixture of pieces from at least four different hearts collected in the same day, which were minced in a blade grinder (Moulinex Moulinette MR, France) to obtain representative samples.

In a second stage, protein extraction processes were carried out. Aiming at performing a RSM analysis, a central composite design (CCD) for two process variables (pH and ionic strength of the extraction buffer), five equidistant levels and five replicates at the central point (pH 6.5 and 2% NaCl) resulted in 13 experiments, which were performed randomly (Table 1). A blend of 4–5 hearts were used in every experiment. The appropriate range of each operational factor was selected according to preliminary factorial experiments carried out in our laboratory: the pH conditions were 4.3, 5.0, 6.5, 8.0, and 8.6, and salt concentrations 0%, 0.58%, 2%, 3.42%, and 4% NaCl, the same as for a previous study on porcine spleens [11].

### 2.3. Microbiological Analysis

Heart samples were serially diluted in sterile tryptone water (tryptone 10 g (Oxoid, L42 Oxoid Ltd., Basingstoke, UK) and NaCl 5 g L^−1^), pour-plated in Petri plates with plate count agar (PCA, Oxoid CM361) as culture medium, and incubated aerobically at 30 ± 1 °C for 72 h [17]. Total aerobic mesophilic bacteria were expressed as log_10_ colony-forming units per g (log_10_ cfu g^−1^). Microbiological analyses were performed the same day of sample collection and preparation.

### 2.4. Protein Extraction

Heart proteins extraction was carried out following the same method as described by Toldrà et al. (2019) [11] with slight modifications. Buffer extraction solutions were prepared with 1:15 M sodium dihydrogen phosphate and 0.1 M citric acid (pH 4.3), 1:15 M potassium dihydrogen phosphate and 1:15 M sodium dihydrogen phosphate (pH 5.0, 6.5, and 8.0), and 0.1 M HCl and 0.1 M Tris (pH 8.6) in distilled water. NaCl was then added to the buffers according to the experimental design.

Five hundred grams of porcine heart, a mixture of pieces from at least four different organs, were introduced into a cutter vessel Sammic CKE-5 (Sammic S.L., Barcelona, Spain) and minced at 2100 rpm for 45 s. Subsequently, the ground sample was mixed, at 1800 rpm for 2 min, with 1000 mL of the corresponding buffer solution according to the CCD, and kept agitated at 300 rpm for 30 min at room temperature. The suspensions were then centrifuged at 20,000× *g* for 15 min at 20 °C in a Sorvall RC-SC plus centrifuge (Dupont Co, Newton, CT, USA), and the soluble fractions obtained by decanting were stored at 5 °C until analysis.

### 2.5. Physicochemical Characterization

#### 2.5.1. Proximate Analysis

Standard methods were used to analyze the proximate composition of porcine hearts and the soluble fractions of each extraction process. Each product was analyzed in triplicate. Moisture and ash contents were determined according to the Association of Official Analytical Chemists (AOAC) methods [18]. Protein content was estimated from the total Kjeldahl nitrogen (TKN × 6.25) [19] by using a digestion block (C. Gerhardt GmbH & Co., Königswinter, Germany) and a distillation unit Büchi K-314 (Büchi Labortechnik AG, Flawil, Switzerland). Total collagen in heart samples was calculated from the hydroxyproline content (Hyp × 8), which was determined through the NMKL–AOAC colorimetric method described by Kolar [20]. It consisted of protein hydrolysis with sulfuric acid, oxidation of hydroxyproline with chloramine-T, and formation of a red-purple complex with p-dimethylaminobenzaldehyde, which was measured at 560 nm by using a Cecil CE 1021 UV-Vis spectrophotometer (Cecil Instruments, Cambridge, UK). Total fat content was determined gravimetrically by Sohxlet extraction [21]. The fat was extracted for 5 h from the previously hydrolyzed and dried sample, using diethyl ether. The extraction solvent was removed through evaporation, and the residue was dried and subsequently weighed after cooling. Atomic emission spectrophotometry (SpectrAA Varian 50B; Agilent Technologies, Palo Alto, CA, USA) with a multielement lamp at a wavelength of 248.3 nm was used to analyze the iron content in aqueous solutions of previously ashed samples.

#### 2.5.2. SDS–Polyacrylamide Gel Electrophoresis (SDS–PAGE)

SDS-PAGE was performed using acrylamide gels with stacking (T 3.94%, C 2.66%) and separating gel (T 15%, C 2.66%) zones, which were prepared with acrylamide:bisacrylamide 40% (37.5:1) solution (Bio-Rad), according to the Laemmli method as described by Fort et al. [22], using a Mini-Protean^®^ 3 electrophoresis system (Bio-Rad Laboratories Inc., Hercules, CA, USA). Gels were run at 70 V for 30 min and then at a constant voltage of 120 V for 45–60 min. The approximate molecular weights were estimated using molecular weights markers from 10 to 220 kDa (BenchMark™ Protein Ladder, Invitrogen, Carlsbad, CA, USA). The gels were fixed with 2.5% gluteraldehyde solution, then stained with Coomassie Blue (0.1% PhastGel Blue R solution in 30% ethanol and 10% acetic acid), distained with 30% ethanol and 10% acetic acid, and preserved in 10% acetic acid and 10% glycerol.

Protein extracts were diluted at a ratio of 1:4 in pH 6.8, 10 mM TrisHCl, 1 mM EDTA, 1% sodium dodecyl sulfate (SDS) and 1% β-mercaptoethanol buffer, and subsequently heated at 100 °C for 5 min and centrifuged at 8000 rpm. Immediately before to the electrophoresis performance, these solutions were diluted to 50% with a buffer containing 1.25 mL Tris HCl 0.05 M pH 8.8, 1% SDS, 2 mL glycerol, and 1.75 mL alcoholic solution of bromophenol blue (0.01%) as tracking dye.

### 2.6. Technofunctional Properties

#### 2.6.1. Protein Solubility

Protein solubility was calculated from the protein content and the corresponding yield of soluble fraction obtained after protein extraction procedure as explained in “Protein Extraction” (Section 2.4), relative to the total protein content of heart.

#### 2.6.2. Foaming Properties

The foaming properties were determined as described in Davila et al. [23]. Three aliquots of 200 mL of protein solutions (5 g L^−1^) of every fraction were prepared in distilled water, and then transferred to 1000 mL volumetric flasks. Solutions were whipped in a Braun Multimix M700 mixer (Braun Española S.A., Barcelona, Spain) with two whisks (Ø = 5 cm) at 1000 rpm for 10 min. The flasks were placed on a rotational plate during mixing to form homogeneous foams. Afterward, the foaming capacity (FC) was determined as the volume (mL) of foam after 2 min at rest. Foam stability was determined using a gravimetric method as follows: measured quantities of foam were carefully placed in three dry stainless steel sieves to let the released liquid drain, and the remaining foam was weighted every 10 min for a period of 60 min. The percentage of remaining foam versus time was plotted, and relative foam stability (RFS), defined as the time (min) needed for the disappearance of 50% of the initial foam, was calculated by fitting data to an exponential decay function y = y_0_ + a × 10^(–bx)^.

#### 2.6.3. Emulsifying Properties

The turbidimetric method reported by Pearce and Kinsella [24] with slight modifications [25] was used to determine the emulsifying properties. Solutions of protein extracts in distilled water were prepared at 5 g L^−1^ of protein. One hundred fifty milliliters of each solution was homogenized along with 50 mL of commercial corn oil using a hand-operated laboratory piston-type homogenizer (MFC MicrofluidizerTM Series 5000, Microfluidics Corporation, Newton, MA, USA) at 12 MPa and 40 L h^−1^ output flow, with recirculation for 90 s. Triplicate preparations were carried out for each sample. The emulsions were diluted 2500-fold with 0.1% sodium dodecyl sulfate (SDS), immediately after homogenization (t = 0) and after 10 min of emulsion rest (t = 10). The absorbance of the diluted emulsions was then determined at 500 nm in a Cecil CE 7400 spectrophotometer (Cecil Instruments Ltd., Cambridge, UK). Each determination was performed in duplicate. Results were reported as emulsifying activity index (EAI) and emulsion stability index (ESI), which were calculated as follows: EAI (m^2^ g^−1^ protein) = 2·T/ϕ·C; where T is turbidity, ϕ is the volume fraction of the dispersed phase, and C is the weight of protein per unit volume of aqueous phase before the emulsion is formed; ESI (min) = T·(Δt/ΔT); where ΔT is the change in turbidity (T) occurring during the interval Δt (between t = 0 and t = 10 min).

#### 2.6.4. Heat-Induced Gelation

The heat-induced gelation capacity of the soluble fractions was tested. Five aliquots of 20 mL of every sample were poured into twist-off glass containers (50 mm in diameter and 20 mm in depth), hermetically closed, and heated for 40 min in a water bath at 80 ± 1 °C. After that, samples were immediately cooled at room temperature. A uniaxial compression test with a TA-XT2 texture analyzer (Stable Micro Systems Ltd., Surrey, UK) was undertaken on the samples in the same receptacle where the gelation was produced. A cylindrical aluminum plunger of 25 mm diameter was used to compress samples until 50% deformation at a rate of 1 mm s^−1^. The strain/distance curve was recorded and the total work involved in performing the test was considered as a measure of gel firmness (N mm). Mean values (*n* = 5) were used for sample comparison.

### 2.7. Statistical Analysis

The SPSS software package version 23.0 (2015) for Windows (IBM Corporation, Armonk, NY, USA) was used for statistical analysis. The experimental data of the protein extracts were fitted to a second-degree polynomial regression model, which included the coefficients of linear, quadratic, and the two-factor interaction effects. Parameters of the second-order polynomial model were estimated using the linear regression procedure with the backward method (inclusion and exclusion criteria at *p* < 0.05 and *p* > 0.1, respectively). Analysis of variance (ANOVA) was performed for the models calculated from the linear regression and the criteria used to evaluate the models were the R^2^ adj value, and significance of the model and the estimated coefficients. Significance was attained for *p* < 0.05. The 3D response graphs of predicted values through the RSM models were plotted using SigmaPlot for Windows v. 11 (2008) (Systat Software Inc., San Jose, CA, USA).

## 3. Results and Discussion

### 3.1. Physicochemical and Microbiological Characterization of Porcine Hearts

Proximate composition, collagen and iron content, weight, pH, color, and microbiological counts from porcine hearts are shown in Table 2. The average weight of the hearts was 407.67 ± 36.14 g and the pH 5.89 ± 0.10, which is in the normal pH range of pork meat (5.6–6.2). Overall proximate composition did not differ much from that reported by Tsermoula et al. [16], except for the slightly higher humidity in our samples and the consequent somewhat lower values of the other components, but leading to similar moisture:protein ratios [16]. Moisture in the heart samples analyzed was also 3.5% higher than that reported by Seong et al. [26], who also found fat contents that doubled our values. Such variability could be explained not only by the different crossbred used in both studies but also by the higher life weights of the pigs, which in the referred work were reported to be about 130–140 kg. The total mesophilic aerobic bacterial counts were in the interval between 3 and 4 log units, in agreement with the values reported elsewhere [27], and within the range of acceptability of the hygienic processing criteria for meat products (Commission Regulation (EC) No. 2073/2005) [28].

### 3.2. Extractability and Compositional Characteristics of Heart Protein Fractions

Table 3 shows the yield of every extraction process and the proximate composition of the soluble extracts as a function of pH and salt concentration of the extraction buffer. A significant increase in viscosity was observed in all solutions after refrigeration at 5 °C, in some cases (all extracts at pH ≥ 6.5) becoming very dense solutions with lumpy gelled appearance.

Concerning the proximate composition of the protein extracts, average contents of 94.9 ± 1.1% moisture, 2.91 ± 0.5% protein, and 1.8 ± 0.75% ashes, were obtained. The NaCl added to the extraction buffer influenced the moisture content of the soluble extracts. As expected, low NaCl entailed a lower ash content and consequently higher relative moisture in the liquid extracts. At any pH, the protein content of the extracts was higher at increasing ionic strength. Although there was not as much clear effect of the pH, for the same NaCl content the highest protein percentage was always found in the extracts obtained at higher pH values.

As can be observed, the average yield of soluble fraction for all extractions was 68.03 ± 1.74; the most distant from the mean value were the extracts obtained at pH 5 and 3.42% NaCl, which showed the minimum yield (59.5%), and at pH 8.6 and 2% NaCl, which reached the maximum (72.82%). The highest values corresponded to the pH axial points, pH 8.6 and 4.3, both containing the salt content corresponding to the central point (2% NaCl). Overall, all processes at pH 6.5 resulted in a yield close to the mean value, regardless of their ionic strength. Nevertheless, the same behavior was found when combining acid pH with low salt or alkaline pH with a considerable amount of NaCl. It is worth noting that the extract obtained by using a buffer at pH 8.6 and 2% NaCl was the one that showed both the best yield and the highest protein content.

Soluble protein recovery was calculated from the protein content of the extracts referred to the corresponding yield of the extraction process and it was reported as protein solubility, that is, percentage of protein in the solutions with respect to the total protein content of porcine heart (Table 4). Solubility ranged from 22.4% (at pH 4.3 and 2% NaCl) to 44.3% (at pH 8.6 and 2% NaCl), confirming the influence of pH in this property. In our heart extracts, the protein contents were somewhat higher while the solubility was slightly lower, as compared to protein extracts from porcine spleen obtained in a previous study, due to the different ratio of solvent to material used in the extraction processes, 10:1 for the spleen proteins and 2:1 for the heart proteins, which result in a higher protein content solution but with a lower solubilization yield [11]. Protein yields around 50% (*w*/*w*) were obtained in several studies on the extraction of water and salt soluble proteins from bovine coproducts [14,29]. According to Selmane et al., yields of protein recovery from pork and beef lungs were between 48 and 55% (*w*/*w*) [30]. Moreover, Tsermoula et al. reported 51.53–55.74% recovery of the total protein from bovine and porcine hearts through alkali and acid solubilization [16].

The quadratic model for solubility was found to be significant (*p* = 0.000) and revealed an adjusted coefficient of determination (R^2^ adj) of 0.978 (Table 5), indicating that close to 98% of experimental data variation is explained by the estimated equation. The proposed quadratic model includes pH and pH^2^, NaCl concentration, and three pH–NaCl interactions. It is widely understood that the solubility of a protein is highly dependent on pH and salt concentration. As shown in the response surface (Figure 3a), higher solubility values correspond to the pH range from 6.5 to 8.5, and solubility improves with salt addition from 1.5 to 3% NaCl, due to an enhancement of myofibrillar proteins solubilization by salt. These results agree with studies on porcine cardiac proteins [15] or proteins extracted from other sources, i.e., porcine spleen [11] or mechanically separated turkey meat [31]. Additionally, ultrasonic treatments were claimed as useful to increase the effective extraction of protein from normal residual meat byproducts such as porcine myocardium without the need for high salt concentrations, as required in conventional extraction methods [15].

### 3.3. SDS-PAGE Profiles

**Figure 1:**
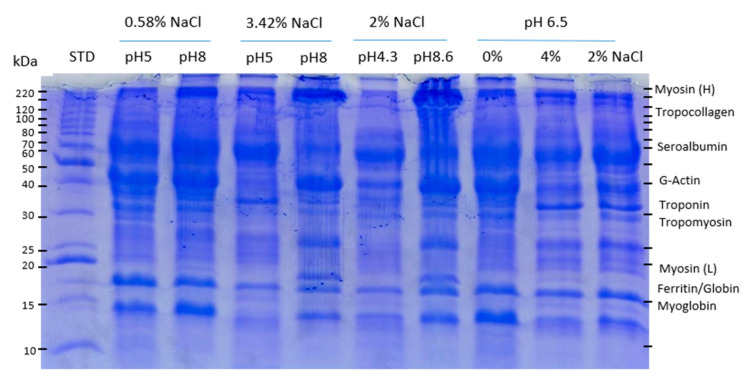
SDS-PAGE (12.5% PA) patterns of soluble proteins extracted from porcine heart as a function of pH and salt concentration of the extraction buffer. STD: molecular weight standard from 10 to 200 kDa.

SDS-PAGE electrophoretograms of soluble protein fractions from porcine hearts are shown in Figure 1. All samples showed broadly similar electrophoretic profiles with bands corresponding to the MW of the major sarcoplasmic and myofibrillar myocardial proteins, as well as blood proteins.

Soluble protein fractions are composed mainly of myofibrillar proteins, actin, and myosin [14,32]. All samples exhibited bands corresponding to heavy (H) and light (L) myosin chains (with a MW of 200–220 and 15–25 kDa, respectively) and bands around 40–50 kDa that could correspond to the globular monomeric form of actin (G-actin) (MW of 42 kDa) [15,33]. Bands in the range between 30 and 40 kDa may be assigned to the regulatory proteins associated with actin, troponin complex, and tropomyosin. Sarcoplasmic proteins are water-soluble globular proteins of relatively low MW (15–20 kDa), consisting mainly of enzymes and hem pigments [29]. Other bands in the range from 20 to 70 kDa can be observed in all samples, probably corresponding to blood proteins, albumin (MW 68–70 kDa), which is the most abundant globular protein in plasma; globulins, a heterogeneous group of globular proteins that include a variety of enzymes, carrier, and antigenic proteins with MW ranging from few to hundreds kDa; and proteins associated to red cell membranes (MW ~ 30–240 kDa) [34,35].

From the main differences among samples, presumably attributable to the pH and ionic strength of the extraction buffer, we can make the following remarks:

Focusing on myosin, the solubility is favored by extraction conditions combining high pH, far from the isoelectric point, together with high ionic strength. This is likely due to alkaline pH loosening the tissues and facilitating structural changes [15]. Samples at pH 8.6 (lane 7) and pH 8 (lane 5) showed the most intense bands of heavy myosin (220 kDa) along with the presence of a band that matches the MW of light myosin (20 kDa). At pH 4.3 and 5 the light chain is not observed and the intensity of the myosin H band is weaker. Increasing the ionic strength of the buffer by the addition of NaCl aids myosin extraction, as confirmed by the fading of the myosin H band and the absence of the myosin L band in samples with low salt content (0.58% NaCl), despite being at pH 8 (lane 3). This effect is also noticeable even when comparing samples at pH 6.5 (lanes 8, 9, and 10), since a slight increase in myosin recovery can be observed as salt concentration increases, in agreement with the protein solubility results. On the other hand, the bands corresponding to the molecular weight of G-actin also show greater intensity in soluble extracts at pH ≥ 5 and seems to be less influenced by NaCl contents.

One band in the range 25–30 kDa can be observed only in some extracts, those corresponding to pH ≥ 6.5 and salt content ≥ 2%. The band could putatively be assigned to low MW salt soluble globulins or several sarcoplasmic enzymes (myokinase, triosephosphate isomerase, phosphoglycerate mutase) [36].

Moreover, in most protein extracts there were two bands in the range of 15–20 kDa MW that follow the same pattern of intensity among the different samples. Looking at their MW, the band of lower MW may correspond to the hemoproteins, myoglobin, and the globin monomer of hemoglobin (16–18 kDa MW approximately), and the other band, with a slightly higher MW (19–21 kDa) to ferritin subunits [37]. The effect of ionic strength on these proteins can be easily observed by comparing the three samples at pH 6.5 (lanes 8–10), the two samples at pH 8 (lanes 3 and 5) or at pH 5 (lanes 2 and 4). The intensities of the bands are always greater in samples with low salt content (0–0.58%) for all pHs. The effect of pH is evidenced from the comparison of 2% NaCl samples at pH 4.3, 8.6, and 6.5 (lanes 6, 7, and 10, respectively). The bands become paler as pH decreases; the highest intensity corresponding to samples at alkaline pH.

Although SDS-PAGE, as carried out in this study, cannot be considered as a quantitative method, it has been shown that differences in polypeptide profiles may be related to the functional properties of protein samples [38].

### 3.4. Effects of the Extraction Conditions on the Functional Properties of Soluble Protein Extracts

The technofunctionality of protein extracts obtained at different extraction conditions are shown in Table 4. Significant second-order polynomial models were obtained for all the response variables (*p* < 0.05) (Table 5). The 3D response surface graphs and contour plots can be found in Figure 3.

#### 3.4.1. Foaming Properties

**Figure 2:**
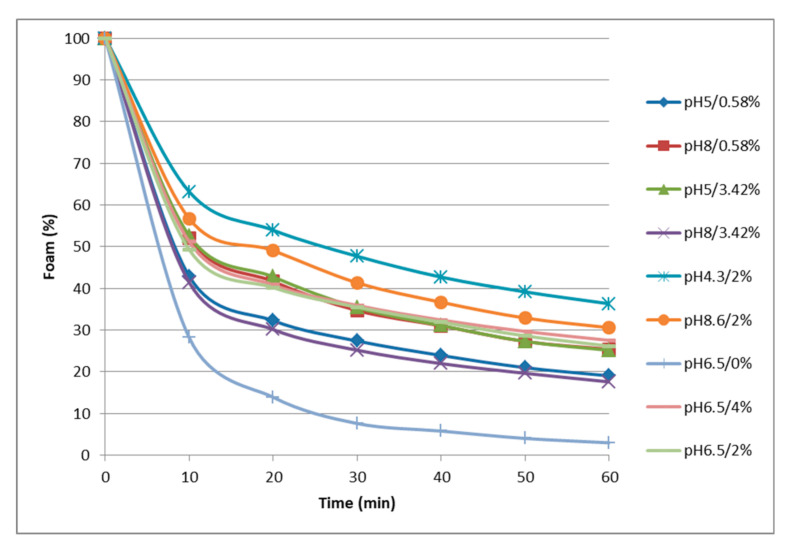
Vanishing kinetics of foam from porcine heart protein extracts (5 g L^−1^), obtained with extraction buffers at different pH and NaCl contents. Points refer to the relative percentage of the initial foam remaining at 10 min intervals.

Proteins are known to enhance and to stabilize foams by adsorbing at the air–liquid interface after undergoing unfolding and molecular rearrangement. Looking at the experimental results on foamability (Table 4), highest foaming capacity (FC) and stability (RFS) corresponded to the solutions extracted with acid buffers. FC values ranged from 283 to 738 mL of foam. It showed low values for solutions at pH ≥ 6.5 and reached higher values at pH 5 (572 mL) and at pH 4.3 (738 mL). At pH 6.5–8.3, a drop of up to about half the volume of foam as compared to the acid extracts was observed. This behavior is in agreement with the one described for other proteins, e.g., globulins from porcine plasma [23]. Concerning the foam stability (Figure 2, RFS values Table 4), the more unstable foam corresponded to the extract recovered at pH 6.5 without added NaCl, and the one showing better stability was again the one produced with the solution of the acid extract (pH 4.3). At pH 5 and 6.5 and using a buffer with increased ionic strength (2–4% NaCl) improved the foam stability of the protein extracts. RFS was also good for solutions at pH 8.6, but this feature is of little relevance due to the fact that it was the extract with the lowest foaming capacity (283.3 mL).

Since the foaming properties of proteins are related to their hydrophobicity and their charge [39], our results could be explained by increased protein surface hydrophobicity at acid pH. The unfolding of protein molecules exposes hydrophobic groups, resulting in increased interaction at the air–water interface. According to Yang et al., the hydrophobicity of myofibrillar proteins decrease as pH increases from 5.0 to 8.0 [40].

**Figure 3:**
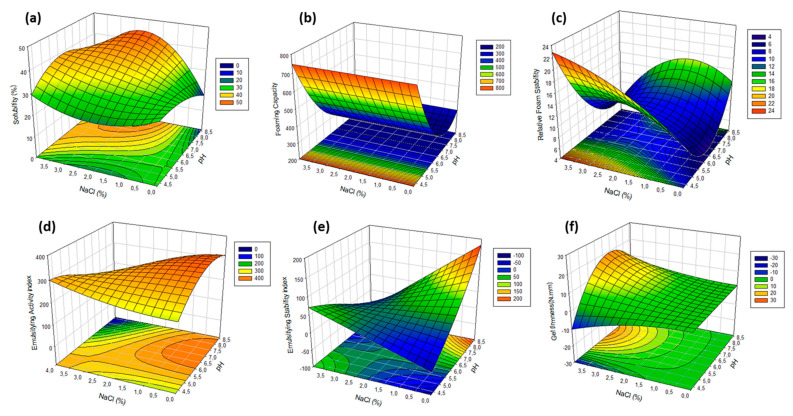
Response surface graphs and contour plot of the protein extracts from porcine heart as a function of pH and salt concentration of the extraction buffer. (**a**) Protein solubility (%) and surface properties (5 g L^−1^ protein); (**b**) foaming capacity (mL); (**c**) relative foam stability (min); (**d**) emulsifying activity index (m^2^ g^−1^); (**e**) emulsion stability index (min); and (**f**) gel firmness after heating (80 °C for 40 min) (N mm).

Table 5 shows the models that better fit to our experimental data on FC and RFS. The response surfaces of both variables are shown in Figure 3c,d, respectively. Regarding to the second-order polynomial functions obtained, the FC response can be explained by the pH of the extraction buffer itself and shows a good fit to the experimental data (R^2^ = 0.901). Conversely, a more complex model that includes NaCl concentration and pH–NaCl interaction was obtained for the RFS variable, although it shows a worse fit to the experimental data (R^2^ = 0.614). As can be seen in Figure 3c, foaming ability reaches a maximum at acidic pH values and worsens with increasing pH, this behavior being irrespective of the NaCl concentration. The model proposes that the FC of 5 g L^−1^ solutions (200 mL) of the water-soluble heart proteins after the whipping process would be good at pH lower than 5.5, with specific foam volumes of 500–750 mL. Surprisingly, the behavior of the FC variable for porcine heart extracts showed to be opposite to the extracts coming from porcine spleen, as reported in Toldrà et al. [11], thus confirming the influence of both the raw material and the extraction process in the technofunctionality of the soluble extracts obtained from different organs [30,41]. Only the heart extracts obtained at acidic pH showed foaming capacity comparable to the extracts from spleen proteins, which had shown to be good at any pH. This behavior can also be related to the solvent:material proportions used to obtain heart extracts; with a ratio of 2:1, a solubilization yield lower than that obtained with the 10:1 ratio used for extractions of soluble spleen proteins. It is possible that the availability of solvent acted as a limiting factor and the proteins with the best surface properties remained in the insoluble residue, even at the most favorable pH and ionic strength conditions for their extraction.

According to the response surface for the variable RFS (Figure 3d), the foam stability can also be considered acceptable at acid conditions and improves with increasing NaCl concentration. The foams show to reach maximum stability at low pH values and high ionic strength, with a linear increase from 0 to 4% NaCl when using an extraction buffer at pH 4.5. The predicted minimum foam stability corresponds to pH near the isoelectric point (*pI*) of myofibrillar proteins (pH 5–5.5). This fact could be a consequence of the protein precipitation, so that there would not be enough protein able to adsorb at the surfaces. Far from this *pI*, proteins with flexible structure form denser and thicker adsorption layers, thus ensuring better stabilization. Added NaCl at acid pH increases further the adsorption and the repulsion between the surfaces (probably by steric and/or osmotic mechanism).

From our results, the best extraction conditions to obtain a soluble protein fraction from porcine hearts with good foaming properties would correspond to acid pH and high salt content.

#### 3.4.2. Emulsifying Properties

The emulsification properties of acid and alkaline extracted proteins were evaluated by their ability to form and stabilize oil-in-water emulsions. EAI indicates the ability of protein to adsorb at the interface during the formation of emulsion, avoiding flocculation and coalescence of the small fat droplets. ESI estimates the decreasing rate of the emulsion turbidity due to fat droplet coalescence and creaming. Therefore, EAI and ESI increase when proteins favor emulsion formation and stabilization, respectively [30].

Experimental results concerning emulsifying properties are shown in Table 4. The mean EAI value for all the protein extracts was 260.3 ± 59.7 m^2^ g^−1^, ranging from 150.4 (pH 8 and 3.42% NaCl) to 354.7 (pH 6.5 without added NaCl). Overall, solutions at pH > 6.5 with low NaCl contents were the ones leading to higher EAI. Steen et al. also found that an increase in salt concentration decreased the emulsifying properties of water and salt soluble proteins from pork liver [29]. Nevertheless, in our results the low emulsifying activity of acid solutions (pH 5) seemed to be somewhat compensated by increasing NaCl. In fact, the impact of salt on the emulsifying properties of proteins, both of animal and vegetable origin, which can be found in the literature leads to contradictory results and diverse mechanisms are proposed to support each finding [29]. Looking at ESI, significantly more stable emulsions were obtained at pH 8 and 0.58% NaCl (170.62 min) as compared to the rest of extracts, which on the whole showed an average ESI value of about 26.43 ± 7.17 min. Dàvila et al. [23] also reported ESI values for porcine plasma globulins and albumin from four- to five-fold higher at pH 7.5 than at lower pH (pH 6.5 and 4.5), which were attributed to the effect of protein aggregation phenomena induced by acidification. However, none of the other alkaline extracts with higher ionic strength allowed for obtaining such stable emulsions.

Regardless of the fractions that provided emulsions with higher indexes, it should be noted that the heart extracts at any condition displayed rather poorer emulsifying properties than extracts from porcine spleen, which were reported to achieve EAI values from 244 to 500 m^2^ g^−1^ and ESI values from 57 to 381 min [11]

Mathematical models for both the emulsifying activity and emulsion stability indexes are shown in Table 5. Emulsion indexes show to be influenced by both pH and ionic strength, but it is worth noting that the models merely account for 65.5% and less than 45% of the data variability for EAI and ESI, respectively. As can be observed from the response surface in Figure 3d, heart protein extracts (5 g L^−1^) would exhibit relatively low emulsifying activity at any pH/NaCl combination, up to 300–400 m^2^ g^−1^ at best, which is much lower than the capacity to form o:w emulsions of blood proteins [23]. In spite of this, it is important to highlight that the EAI values obtained were similar to those displayed by palm seed protein extracts [42], and even higher than those from ovine whey proteins [43], fish protein extracts [44], yeast proteins, and egg albumins [24].

#### 3.4.3. Gelling Properties

Gelation is as a multistage process involving the initial denaturation of native protein structure and unfolding of the protein molecules, followed by aggregation and cross-linking between proteins. Since heating induces protein denaturation, aggregation, and eventually the formation of a gel that gives consistency to the food product, many proteins act as gelling or thickening agents when a thermal treatment is applied [45]. Indeed, one of the important functional properties of muscle proteins is their ability to form a gel. Different contribution to both gel formation and gel characteristics have been reported for sarcoplasmic and myofibrillar proteins. For myofibrillar individual proteins such as myosin and protein complexes such as actomyosin, pH significantly influences gel formation. Optimal pH for gelation depends on the concentration of myosin and actin in solution. The state of meat protein molecules, which is influenced by the ionic strength, is also important for gelling capacity since gel formation results from protein–protein and protein–solvent interactions [46].

Although salt-soluble muscle proteins are known to contribute primarily to gel formation, their recovery from some organs is difficult due to the high content of connective tissue [7]. All extracts contain myofibrillar proteins, as shown by SDS-PAGE, but their relatively low protein content has probably impaired the heat-induced gel forming ability. Nonetheless, it is interesting to realize (Table 4 and Figure 3f) that some of them displayed heat-induced gelling capacity in spite of their relatively low protein content, which would probably improve after a gentle concentration process.

Protein extracts at pH ≤ 5 and pH 8.6 showed some aggregation after the heat treatment, but they were not able to form consistent gels. On the other hand, self-supporting gels were obtained on heating extracts at pH 6.5–8, which showed improved firmness with increasing NaCl concentration. It is worth noting that all heart protein gels obtained in the present work showed high syneresis. Addition of NaCl has been described as a key requirement for good gelling properties for myofibrillar protein-rich extracts prepared from bovine hearts using a surimi-like process [47]. Tsermoula et al. also reported an increase in gel hardness when 2% NaCl was added to the extracts from porcine heart standardized at 8% protein content [16]. Nevertheless, gel forming ability of water soluble pork liver proteins without salt showed to be higher compared to high salt concentrations [29]. It seems interesting to highlight that extracts able to form firm gels somewhat coincide with the ones showing the band in the range 25–30 kDa of their electrophoretic profile, which supposedly could correspond to several blood globulins. Gelation studies on blood proteins reported that the development of strong gels in a wide range of pH was mainly attributed to the globulins fraction that were also responsible for the strength of gels when mixed with albumin [23,48]. Likewise, Steen et al. observed that higher salt concentrations shifted onset gelation temperature to lower temperatures [29].

The mathematical model obtained (Table 5) fits well to the experimental data (R^2^ = 0.946) and confirms the strong dependence of the gelling capacity on the ionic strength, as well as its interaction with the pH. As can be seen in Figure 3f, the effect of pH is only important when the extraction process is carried out with buffers with high NaCl content. Higher gel firmness values correspond to solutions at pH 6–7.5, showing maximum values at 3–4% NaCl. At lower NaCl contents, the firmness is reduced in half and becomes practically independent of the pH factor.

## 4. Conclusions

The protein functional properties strongly depend on the extraction conditions, which affect each property in a different way. Protein extracts showing diverse functionality were obtained from porcine hearts by using different extraction buffers. The conditions that showed both the best extraction yield and the highest protein content were pH 8.6 and 2% NaCl. Highest foaming capacity and stability corresponded to the solutions extracted with acid buffers. The foam stability improved with increased ionic strength. The highest emulsifying activity was found in solutions at pH > 6.5 with low NaCl, whilst the more stable emulsions were obtained at pH 8 and 0.58% NaCl; nevertheless, heart protein extracts showed rather poorer emulsifying properties than extracts from other porcine organs. Although protein extracts at pH ≤ 5 and pH 8.6 were not able to form consistent gels, self-supporting gels were obtained from extracts at pH 6.5–8, all showing enhanced firmness as NaCl concentration increases.

Response surface methodology has been used successfully to systematically study the effects of extraction conditions on the recovery and functionality of soluble proteins from porcine hearts. The mathematical models obtained allow for knowing the variables that influence every functional property and their interactions, and also to determine the best conditions to obtain ingredients with a specific functionality. Therefore, these models can be used to foreknow the most suitable operating conditions according to the intended use of the protein extracts.

Recovering technofunctional proteins for food applications from industrial byproducts would contribute to improving the sustainability of the meat industry.

## Figures and Tables

**Table 1:** Experimental design for the optimization of the recovery technofunctional protein extracts from porcine hearts, according to the central composite design (CCD).

Run	Coded Variables		Order	Experimental Variables	
				pH	NaCl (%)
1	−1	−1	13	5	0.58
2	+1	−1	5	8	0.58
3	−1	+1	7	5	3.42
4	+1	+1	12	8	3.42
5	−α	0	6	4.3	2
6	+α	0	1	8.6	2
7	0	−α	3	6.5	0
8	0	+α	10	6.5	4
9	0	0	4	6.5	2
10	0	0	9	6.5	2
11	0	0	11	6.5	2
12	0	0	8	6.5	2
13	0	0	2	6.5	2

Coded variables: 1 = high factor level, −1 = low factor level, 0 = central point; negative and positive default α-values indicate the low and high axial levels, respectively (α = 1.414).

**Table 2:** Physicochemical characteristics and microbiological quality of raw porcine hearts (means ± SD, *n* = 6).

Weight (g)	407.67 ± 36.14
pH	5.89 ± 0.10
Moisture (%)	79.31 ± 0.34
Protein (%)	16.70 ± 0.18
Collagen (%)	1.13 ± 0.30
Fat (%)	2.46 ± 0.54
Ashes (%)	1.04 ± 0.07
Iron (ppm)	60.33 ± 20.19
*Color properties*	
Chroma	19.79 ± 0.59
Hue (°)	25.60 ± 1.39
Lightness (L*)	32.55 ± 1.16
Bacterial counts (log cfu g^−1^)	3.49 ± 0.46

**Table 3:** Yield and proximate composition of soluble protein fractions from porcine heart as influenced by pH and NaCl content (%) of the extraction buffer.

pH	NaCl	Yield (%)	Proximate Composition (%)		
			Moisture	Protein	Ash
**5**	0.58	67.2	96.63	2.33	0.89
**8**	0.58	63.2	95.57	2.85	1.11
**5**	3.42	59.5	93.78	3.03	2.74
**8**	3.42	68.6	94.21	3.35	2.00
**4.3**	2	71.3	95.63	1.79	2.01
**8.6**	2	72.8	94.61	3.59	1.62
**6.5**	0	68.6	96.82	2.24	0.37
**6.5**	4	67.7	92.84	3.35	3.30
**6.5**	2	68.0 ± 1.7	94.64 ± 0.39	1.88 ± 0.20	3.05 ± 0.01

Central point (pH 6.5 and 2% NaCl); mean ± SD (*n* = 5).

**Table 4:** Technofunctional properties of soluble protein fractions from porcine heart as influenced by pH and NaCl content (%) of the extraction buffer.

pH	NaCl	Solubility (%)	Foaming		Emulsifying		Gelling
			FC	RFS	EAI	ESI	GS
5	0.58	26.9	596.5	8.53	236.44	20.13	1.31
8	0.58	29.9	375.9	12.37	297.32	170.62	2.18
5	3.42	31.5	572.0	12.89	278.89	41.10	1.19
8	3.42	38.9	343.2	8.03	150.39	22.47	11.47
4.3	2	22.4	738.1	22.04	259.09	25.65	0.82
8.6	2	44.3	283.3	16.17	251.79	19.94	2.42
6.5	0	26.3	365.0	5.55	354.74	35.19	2.15
6.5	4	39.2	324.1	11.64	206.35	26.15	18.77
6.5	2	35.8 ± 1.1	326.8 ± 41.6	11.15 ± 1.78	308.00 ± 39.01	25.32 ± 6.50	5.38 ± 0.89

FC: foaming capacity (mL). RFS: relative foam stability (min). EAI: emulsifying activity index (m^2^ g^−1^). ESI: emulsifying stability index (min). GS: gel firmness (N mm). Central point (pH 6.5 and 2% NaCl); mean ± SD (*n* = 5).

**Table 5:** Polynomial models for the response surface methodology (RSM) optimization of functionality of protein extracts from porcine heart as a function of pH and salt concentration of the extraction buffer.

	Protein Solubility (%)	Foaming		Emulsifying		Gelling
		FC	RFS	EAI	ESI	GS
Coefficients						
Constant	14.762	2732.309	64.900	−385.954	−110.123	1.629
pH	6.090	−647.477	−18.880	201.037	ns	ns
NaCl	−16.740	ns	4.396	ns	55.987	ns
pH*NaCl	ns	ns	ns	ns	ns	ns
pH^2^	−0.669	43.023	1.502	−13.923	3.971	ns
NaCl^2^	ns	ns	ns	28.200	ns	−11.310
pH^2^*NaCl	0.550	ns	ns	ns	−1.576	ns
pH*NaCl^2^	1.408	ns	ns	−5.456	ns	3.544
pH^2^*NaCl^2^	−0.236	ns	−0.021	ns	ns	−0.253
Adjusted R^2^	0.978	0.901	0.614	0.655	0.490	0.946
Significance	0.000	0.000	0.017	0.011	0.028	0.000

FC: foaming capacity (mL); RFS: relative foam stability (min); EAI: emulsifying activity index (m^2^ g^−1^); ESI: emulsifying stability index (min); GS: gel firmness (N mm); ns: not significant (variable not included in the model).
